# Computational Adaptive Optics for HAR Hybrid Trench Array Topography Measurement by Utilizing Coherence Scanning Interferometry

**DOI:** 10.3390/s25134085

**Published:** 2025-06-30

**Authors:** Wenyou Qiao, Zhishan Gao, Qun Yuan, Lu Chen, Zhenyan Guo, Xiao Huo, Qian Wang

**Affiliations:** 1 School of Electronic and Optical Engineering, Nanjing University of Science and Technology, Nanjing 210094, China; qiaowenyou@163.com (W.Q.); guozy15@njust.edu.cn (Z.G.); huoxiao111233@gmail.com (X.H.); wangqian47@njust.edu.cn (Q.W.); 2 Jiangsu Energy Measurement Data Center, Jiangsu Institute of Metrology, Nanjing 210023, China; june_0623@126.com

**Keywords:** coherence scanning interferometry, high aspect ratio, sample-induced aberrations, virtual phase filter, aberration correction, topography

## Abstract

High aspect ratio (HAR) sample-induced aberrations seriously affect the topography measurement for the bottom of the microstructure by coherence scanning interferometry (CSI). Previous research proposed an aberration compensating method using deformable mirrors at the conjugate position of the pupil. However, it failed to compensate for the shift-variant aberrations introduced by the HAR hybrid trench array composed of multiple trenches with different parameters. Here, we propose a computational aberration correction method for measuring the topography of the HAR structure by the particle swarm optimization (PSO) algorithm without constructing a database and prior knowledge, and a phase filter in the spatial frequency domain is constructed to restore interference signals distorted by shift-variant aberrations. Since the aberrations of each sampling point are basically unchanged in the field of view corresponding to a single trench, each trench under test can be considered as a separate isoplanatic region. Therefore, a multi-channel aberration correction scheme utilizing the virtual phase filter based on isoplanatic region segmentation is established for hybrid trench array samples. The PSO algorithm is adopted to derive the optimal Zernike polynomial coefficients representing the filter, in which the interference fringe contrast is taken as the optimization criterion. Additionally, aberrations introduce phase distortion within the 3D transfer function (3D-TF), and the 3D-TF bandwidth remains unchanged. Accordingly, we set the non-zero part of the 3D-TF as a window function to preprocess the interferogram by filtering out the signals outside the window. Finally, experiments are performed in a single trench sample and two hybrid trench array samples with depths ranging from 100 to 300 μm and widths from 10 to 30 μm to verify the effectiveness and accuracy of the proposed method.

## 1. Introduction

The structural parameters of finished products of HAR microstructure do not conform to the design specifications, and it directly impacts the quality and performance of micro-nano devices [1,2,3,4]. Coherence scanning interferometry (CSI) [5,6] is a non-destructive measurement technology that can obtain the three-dimensional (3D) topography of microstructures, but the HAR structure of silicon blocks white light and modulates detection light, then introduces aberrations to decrease the diffraction-limited resolution of CSI instrument, expand the interference fringe, reduce the signal-to-noise ratio of the interference signal, and affect the accurate capture of the bottom topography of structure [7,8]. Adaptive optics [9,10,11] techniques can restore ideal imaging performance by measuring and correcting aberrations by hardware components. Thus, our team has proposed a sample-induced aberration compensatable near-infrared CSI to measure the 3D topography for HAR microstructures [12]. This method needs to build an aberration detection module in the optical system to detect pupil aberrations and an aberration-compensating device (a deformable mirror) to compensate for sample-induced aberration. However, this method cannot accurately measure a hybrid trench array with shift-variant aberrations.

Compared with hardware-based wavefront sensing and correction, computational adaptive optics (CAO) is a simple and effective back-end aberration correction technique implemented without needing to employ any extra expensive optics and modify the optical system [13,14,15]. A proposal is made to construct the 3D-PSF model for numerically correcting the raw interference signal affected by aberrations through deconvolution. Although this method can be used to resolve the shift-variant aberrations, it relies on the PSF database built with simulation results from a large number of different parameter HAR structures, and the PSF database building is time consuming. Concurrently, the method also needs to calculate the initial values of HAR trench width and depth from the raw interference signal collected by experiments as the prior knowledge, which is used for matching the 3D-PSF in the database. This process undoubtedly increases the processing flow of this scheme [16]. Furthermore, the pupil function can be expressed as a linear combination of Zernike polynomials, and the pupil function can be optimized using the evaluation function to eliminate the effect of shift-variant aberrations on imaging. Adie et al. proposed optimizing the pupil function by changing the coefficients of Zernike polynomials, while monitoring the trend of the evaluation function [17]. However, this method cannot achieve automatic correction. Pande et al. proposed the elastic backpropagation algorithm, which can automatically correct image aberrations without obtaining specific parameters of the imaging system [18]. However, the algorithm relies on the differentiable properties between the evaluation function and the coefficients of Zernike polynomials, so the choice of the evaluation function is somewhat limited. It should be noted that the key to optimizing pupil function is to estimate the coefficients of Zernike polynomials both rapidly and accurately. Searching orders of Zernike polynomials is inconclusive for different applications [19,20], and its accuracy and efficiency are affected by the searching boundary and step for each coefficient. Meanwhile, the computing complexity of algorithms will result in slow convergence.

The particle swarm optimization algorithm (PSO) jointly proposed by Kennedy and Eberhart is a random search algorithm developed by simulating the foraging behavior of birds [21,22]. The solution to each optimization problem can be regarded as a “particle.” According to the target function for optimization, the fitness of each particle can be calculated. By comparing the current fitness with the historical optimal fitness of the population, the “particles” can carry out the optimal search in the solution space. Currently, the PSO algorithm is widely used in various fields, such as fusion classification, image segmentation, system identification, and neural network training, due to its fast convergence characteristic, and the parameter setting of the algorithm model is simple [23,24]. Facing the sample-induced aberration generated in the measurement of HAR microstructures utilizing CSI, each type of aberration is regarded as a “particle,” and the convergence advantage of the particle swarm algorithm is applied to the aberration correction in the interferogram.

Here, we propose computational adaptive optics for measuring the topography of the HAR structure based on the PSO algorithm. Because the sample topography construction is based on the interference fringe signal of CSI, the interference image affected by aberration is preprocessed by constructing a window function in the spatial frequency domain to retain only the interference signal. For the shift-variant aberrations introduced by the HAR hybrid trench array, each trench under test can be considered as a separated isoplanatic region, and a multi-channel aberration correction scheme based on isoplanatic region segmentation is established. For each isoplanatic region, a virtual phase filter constructed through a combination of Zernike polynomials is employed to eliminate the aberrations, and the PSO algorithm is adopted to derive the optimal Zernike polynomial coefficients representing the filter, in which the interference fringe contrast is taken as the optimization criterion. Finally, the topography of the hybrid trench array is constructed based on the corrected interference signals.

## 2. Methods

### 2.1. Influence of Sample-Induced Aberrations on PSF

The resolution reflects the ability of an optical system to distinguish the details of objects. The optical system images a geometric point as a dispersed spot, and the larger the dispersed spot, the poorer the resolution of the system. The response of the optical system to point objects can be described using the point spread function, which is a mathematical expression of the degree of resolution degradation. Based on the physical concept of PSF, the two-dimensional point-spread function (2D-PSF) of the CSI system can be defined as the combination of a point object and its corresponding point on the reference mirror onto the image plane. The CSI measurement of surface topography follows a linear Fourier model [25,26]. In essence, the PSF provides the interferogram that would be observed if a CSI instrument measures a point-like object. However, aberrations degrade the PSF by adding additional distorted phases $\varphi_{a}$ in the pupil function, resulting in decreased system resolution, while also impairing the ability of CSI to measure steep surfaces [27,28,29].

**Figure 1:**
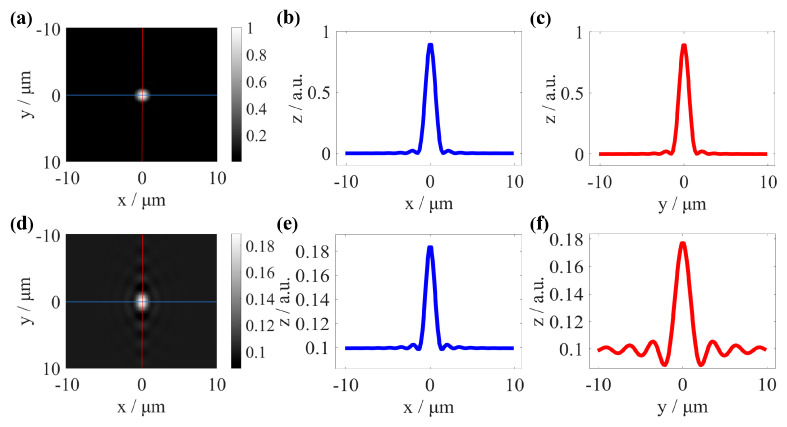
The 2D-PSF of an ideal system and a system with introduced aberrations. (**a**–**c**) The PSF of an ideal system. (**d**–**f**) The PSF of a system with introduced aberrations.

Here, the angular spectrum method (ASM) is utilized to simulate the 2D-PSF at the focal plane of the CSI instrument. The specific modeling process is described in detail in [30], but its detailed explanation is omitted within this context. Figure 1 shows the PSF of an ideal system and a system with introduced aberrations. When pupil aberrations emerge within the test arm of the CSI system, the dispersed spot at the focal plane enlarges. The enlarged dispersed spot leads to a decrease in the resolution of the CSI system. This severe distortion also causes a decrease in the signal-to-noise ratio of the CSI signal and widens the interference fringe [16,30], significantly affecting the ability of CSI to measure the topography of HAR microstructures. The sample-induced aberrations are flexible for HAR structures with different parameters. Therefore, analyzing sample-induced aberrations and establishing an aberration correction method for the fast non-destructive measurement of HAR microstructures is an urgent problem.

### 2.2. Modeling of Derived Aberrations Caused by HAR Hybrid Trench Array

**Figure 2:**
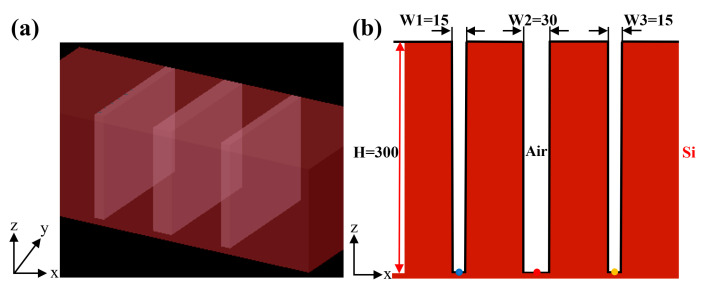
HAR hybrid trench array (sample 2). (**a**) 3D model schematic diagram (red is silicon). (**b**) The x–z cross-sections of HAR hybrid trench array.

In previous studies, we selected HAR single-trench samples with different parameters and HAR trench array samples with a duty ratio equal to 0.5, and the studied samples introduced modulated aberrations at the pupil of the CSI system testing arm [12,30]. Aberration primarily presents as defocus and astigmatism, and the numerical magnitude of aberration at various points within the trench bottom is relatively consistent. The PSF is most often considered to be shift-invariant, and the aberration introduced by the above-mentioned sample is treated with shift-invariant characteristics. Here, we employ a consistent approach [30] to simulate the aberrations induced by the HAR hybrid trench array with a duty ratio that is not equal to 0.5; the sample is shown in Figure 2. The silicon-based HAR hybrid trench array is composed of three trenches, with widths of 15 μm, 30 μm, and 15 μm and depths of 300 μm each, and the spacing between adjacent trenches is 80 μm. The simulation utilizes incident light with a wavelength of 1.32 μm and a numerical aperture (NA) of 0.5. The focus of the incident light is designed at the center point (blue dot, red dot, and yellow dot) of the three trenches. The finite-difference time-domain (FDTD) method is employed to numerically solve Maxwell’s equations for computing the electric field on the surface of the HAR hybrid trench array. Subsequently, the electric field is propagated to the pupil surface using ASM. A parallel methodology is utilized to simulate the y-z cross-section, resulting in the acquisition of two orthogonal light field vectors. Finally, the Zernike wavefront is fitted using the least squares technique to determine the pupil aberrations.

**Figure 3:**
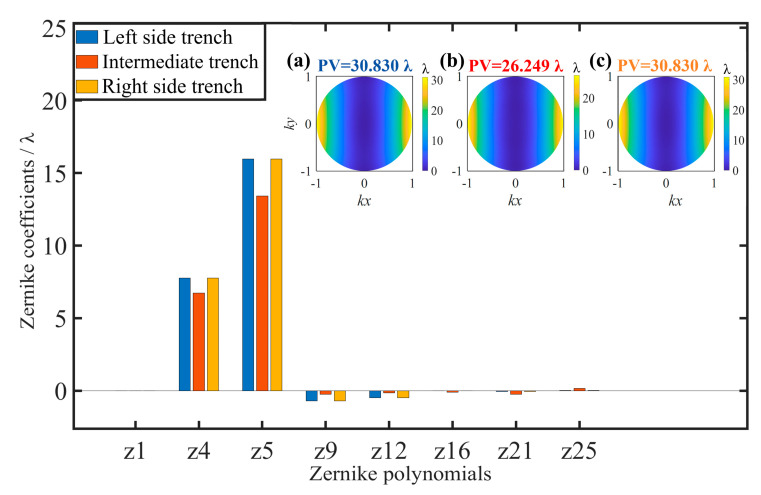
Simulation results of the Zernike coefficients of the pupil aberrations and the wavefront distribution. (**a**) The wavefront distribution with left side trench. (**b**) The wavefront distribution with intermediate trench. (**c**) The wavefront distribution with right side trench.

In Figure 3, it can be observed that the aberrations stemming from the HAR hybrid trench array primarily consist of low-order aberrations, like defocus and astigmatism, a finding consistent with previous simulation results. When the focus of the incident light is designed at the center of the left and right trenches, the corresponding Zernike coefficient values and wavefront PV values of the simulated aberrations are consistent. In contrast, when the focus of the incident light is designed at the center of the intermediate trench, the corresponding Zernike coefficient values of the simulated aberrations and the wavefront PV values change significantly, and the difference value of the wavefront PV is greater than 4.5 wavelengths, indicating that the magnitude of aberrations is primarily determined by the trench structure parameters. Therefore, in the process of CSI measurement, the aberration in the field of view corresponding to each trench in the HAR hybrid trench array is primarily composed of the aberration caused by the trench itself, and aberrations in imaging exhibit characteristics of shift variation for the hybrid trench array sample.

Due to the significant differences in aberrations introduced by the trenches with different parameters, for the HAR hybrid trench array, the modulation aberration introduced by the HAR hybrid trench array cannot be deemed approximate to that of a single trench, and the PSF of the system can be regarded as shift-variant. At this moment, the previously designed near-infrared active compensation CSI system cannot achieve precise compensation and may encounter over-compensation and under-compensation, seriously affecting the accuracy of the CSI measurements of HAR structure topography.

According to the previous simulation results of aberrations, in the field of view corresponding to the bottom of a single trench, the aberrations of each sampling point are basically unchanged [12]; meanwhile, the aberration in the field of view corresponding to each trench in the HAR hybrid trench array is primarily composed of the aberration caused by its structure, and the coherent imaging system in its isoplanatic region is a linear light intensity system with invariance in space. Therefore, each trench under test can be considered as a separated isoplanatic region; we partition the hybrid trench array interference image captured by the CCD into multiple individual trench images with an isoplanatic region and perform aberration correction on each image. 

### 2.3. Principle of CAO

The principle of computational adaptive optics (CAO) has been reported [15]. Here, it is summarized in combination with the coherent imaging theory. The interferogram can be regarded as the convolution result of the objective function and the system PSF, and the PSF is the inverse Fourier transform of the pupil function p(u,v). For a system with aberrations, it is considered that the aberration phase $\varphi_{a}(u,v)$ is superimposed on the wavefront of the ideal system pupil, and the aberrated image $G_{a}(u,v)$ in the pupil plane can be expressed as:

(1) $$G_{a}(u,v)=O(u,v)p(u,v)\exp[i\varphi_{a}(u,v)],$$

where O(u,v) is the Fourier transform of the objective function, and (u,v) are the coordinates of the pupil plane.

According to the principle of phase conjugation, when $\varphi_{a}(u,v)$ is estimated, the aberration of the interferogram $G_{a}(u,v)$ can be eliminated, and the aberration-corrected interferogram $g_{c}(x,y)$ in the image plane is given by:

(2) $$g_{c}(x,y)=F^{-1}\left\{G_{a}(u,v)\exp[-i\varphi_{a}(u,v)]\right\},$$

where (x,y) are the cartesian coordinates of the image plane. $\exp[-i\varphi_{a}(u,v)]$ is referred to as a virtual phase filter, and the aberration phase $\varphi_{a}(u,v)$ is represented by a sum of weighted Zernike polynomials:

(3) $$\varphi_{a}(u,v)=CZ(u,v),$$

(4) $$C=\left[\begin{array}{cccc} c_{1} & c_{2} & \cdots & c_{m} \end{array}\right],Z=\left[\begin{array}{c} z_{1}\\ z_{2}\\ \vdots \\ z_{m} \end{array}\right],$$

where ***C*** is the coefficient matrix of wavefront distortion, ***Z*** is the Zernike polynomials, and *m* is the number of Zernike terms. The coefficient matrix ***C*** is estimated using the interference fringe contrast as the evaluation function for aberration correction. When the interference fringe contrast reaches the maximum, the aberrations are accurately estimated, and the computational aberration correction of the interferogram is achieved. Therefore, the aberration correction of CSI interference images is then transformed into using CAO combined with optimization algorithms to find the optimal coefficients C of Zernike polynomials.

### 2.4. PSO Algorithm

According to the CAO principle, aberration correction introduces a virtual phase filter $\varphi_{a}(u,v)$ into an image with aberrations in the Fourier domain and then obtains the corrected interference fringe image $g_{c}(x,y)$. As introduced in the preceding section, the phase filter $\varphi_{a}(u,v)$ is composed of Zernike polynomials, and the aberrations caused by the HAR structure are mainly defocus and astigmatism. Defocus and astigmatism are both independent and jointly affect the phase variation of interference fringes. Therefore, we can further simplify the construction of the phase filter $\varphi_{a}(u,v)$, transforming the correction of aberrations into the problem of finding the optimal values of the coefficients for defocus and astigmatism.

The PSO algorithm is a model-free optimization technique suitable for complex systems with multiple variables because of its superior optimization ability and fast convergence. In this algorithm, the flock model is abstracted as a “particle” model without mass and volume. The movement speed and direction of each “particle” are influenced by itself and the historical optimal information of the population, thereby completing the optimal exploration of the “particles” in the solution space. Therefore, when solving the phase filter $\varphi_{a}(u,v)$ in this article, the defocus and astigmatism coefficients in Zernike can be used as “particles” for solving. The particle swarm optimization algorithm must be utilized to perform aberration correction on the interference fringe image of the CSI system. The specific process is as follows:

Assuming a coefficient search space in D dimensions, where the values of the coefficients of Zernike polynomials are considered as particles, a population of *N* particles is composed, with the *i*-th particle represented as a D-dimensional vector, and $C_{d}$ representing the value of the *d*-th particle, modeled as the coefficients corresponding to Zernike polynomials.

(5) $$C_{i}=(c_{i1},\;c_{i2},\ldots ,\;c_{iD}),\;i=1,2,\ldots N,$$

The velocity of the *i*-th particle is also a D-dimensional vector, called a step length, expressed as:

(6) $$V_{i}=(v_{i1},\;v_{i2},\ldots ,\;v_{iD}),\;i=1,2,\ldots N.$$

**Figure 4:**
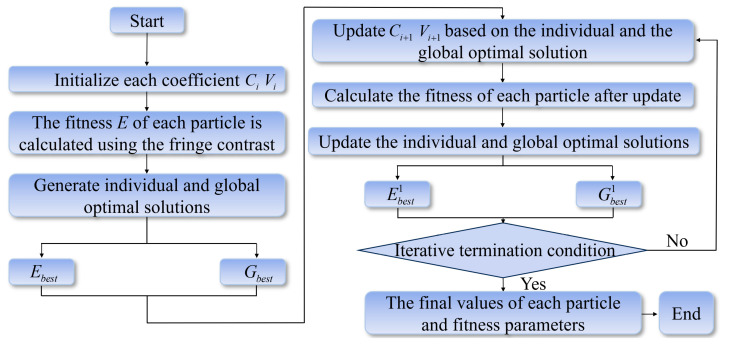
Flowchart of the PSO algorithm.

In order to prevent coupling between the correction terms of Zernike wavefront aberrations, individual best and global best solutions are considered separately. The best position currently searched by the *i*-th particle is referred to as the individual best solution, denoted as:

(7) $$E_{best}=(c_{e1},\;c_{e2},\ldots ,\;c_{eD}),$$

while the global best solution searched by the entire particle swarm is:

(8) $$G_{best}=(c_{g1},\;c_{g2},\ldots ,\;c_{gD}),$$

the step length and coefficients are updated using the individual best solution and global best solution, denoted as:

(9) $$V_{i+1}=W\times V_{i}+\xi_{1}\times R_{1}\times (E_{best}-C_{i})+\xi_{2}\times R_{2}\times (G_{best}-C_{i}),$$

(10) $$C_{i+1}=C_{i}+V_{i},$$

where W is the inertia constant, set to 1; $R_{1}$ and $R_{2}$ are random numbers in the range [0, 1]; $\xi_{1}$ is the acceleration constant, mainly controlling the influence of the individual best solution on updating the iteration step length, and is set to 1; $\xi_{2}$ is the learning rate, mainly reflecting the influence of the global best solution on seeking the solution value, and is set to 1. Therefore, the PSO algorithm is used to estimate wavefront distortion, and then, the aberrations are corrected directly by updating the pupil function in this paper. The PSO algorithm process is illustrated in Figure 4.

### 2.5. Separated the Interference Signal by a Window Function

The light intensity captured by the image plane can be described as the coherent combination of the test light and the reference light, depicted in Equation (11):

(11) $$I_{\lambda }(r,\lambda )={\left|U_{t}(r,\lambda )\right|}^{2}+{\left|U_{r}(r,\lambda )\right|}^{2}+U_{t}(r,\lambda )U_{r}{\left(r,\lambda \right)}^{*}+U_{t}{\left(r,\lambda \right)}^{*}U_{r}(r,\lambda ),$$

where $U_{t}(r,\lambda )$ and $U_{r}(r,\lambda )$ are the image plane test light field and reference light field, respectively. The initial two components of the equation are labeled as the direct current (DC) terms, denoting the intensities of the test light and reference light individually. The final two components, termed the alternating current (AC) terms, represent the interference fringe signals. The interference fringe as a carrier-frequency signal on a CSI image is also affected by aberration [16]. Considering the construction of the surface topography of the sample is based on interference fringe signals, the interferometric signal and the microscopic intensity signal can be separated in the spatial frequency domain. Therefore, the interference signal can be separated by constructing a 3D window function that is cross-sectional like an “umbrella” [31]. This section will focus on describing the origin and generation process of the frequency domain 3D window function used to separate interferometric signal terms.

**Figure 5:**
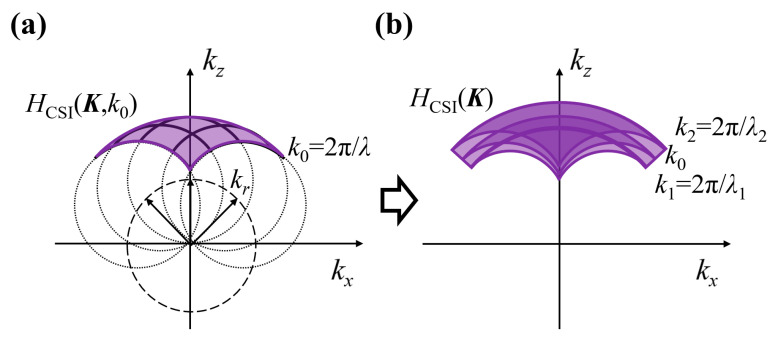
The general 3D pupil function of microscopic interference distribution on the Ewald sphere. (**a**) 3D-TF corresponds to a monochromatic light. (**b**) Low coherence interference 3D-TF corresponding to broadband light.

McCutchen derived that the 3D optical pupil function of a finite NA optical system is a truncated Ewald sphere, known as the McCutchen sphere [29], with non-zero values only on the sphere shell. An optical system’s 3D transfer function (3D-TF) is equivalent to the coordinate-scaled 3D pupil function. According to the research of Coupland et al. [25], under monochromatic light, the transfer function of the interferometric system is equal to the non-interferometric system transfer function convolved with itself in the spatial frequency domain, as shown in Figure 5a, where $k_{r}$ represents the wave vector of the reference light. The distribution of the 3D-TF on the Ewald sphere [32] unveils the signal transmission capabilities of the CSI system in the frequency domain. $H\left(K,k_{0}\right)$ represents the 3D-TF of the system under single-wavelength $\lambda_{0}$ conditions, i.e., the CTF, where K is the spatial frequency vector, and $k_{0}$ is the wave number.

Under the condition of low-coherence microscopic interference with broadband light, the response of the interference system to low-coherence illumination is simply the incoherent superposition of its spectral components. Therefore, the TF of a low-coherence microscopic interference system is given by:

(12) $$H_{CSI}\left(K\right)=\int_{k_{1}}^{k_{2}}S\left(k\right)H_{CSI}\left(K,k\right)dk,$$

where $k_{1}$ and $k_{2}$ represent the wavenumber corresponding to the spectral boundary wavelength, which is also the Ewald sphere radii of the respective monochromatic light; the Ewald sphere radii corresponding to different wavelengths are different, and the distribution area of the general 3D pupil is also different. The distribution of the 3D pupil for broadband light is the superposition of the pupil of all monochromatic light. The schematic diagram of the non-zero part of the TF of the low-coherence microscopic interference system is shown in Figure 5b. It can be seen that the non-zero part of the 3D-TF occupies a specific range in the spatial frequency domain, which represents the frequency bandwidth that the interference signals can transmit. Only the surface information of the sample with frequencies within the bandwidth of the 3D pupil can be transmitted to the image plane.

When the system has aberrations, the 3D-TF or pupil function of the system is no longer in the ideal form. Phase distortion is introduced within the frequency bandwidth. Although aberrations do not change the shape of the pupil or the frequency bandwidth of the TF, the introduced phase distortion will modulate the information transmitted by the system, thus affecting the 3D-PSF. The non-zero part of the aforementioned 3D-TF within the spatial frequency domain represents the distribution range of the interference signal, and its cross-sectional shape resembles an “umbrella”. Therefore, we can design the non-zero part of the 3D-TF as a window function, filtering out the signals outside the window to obtain the interference fringe signals that need to be corrected in this article.

**Figure 6:**
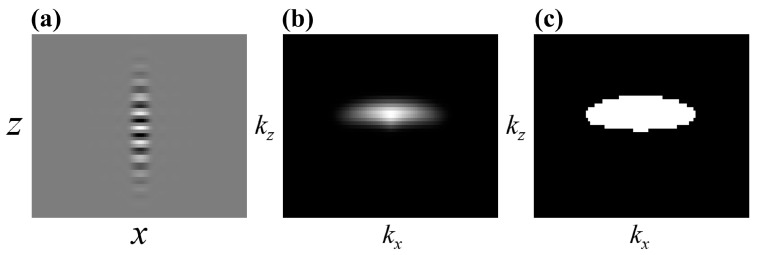
The cross-section images of the system’s PSF, TF, and window function without aberrations. (**a**) PSF. (**b**) TF. (**c**) Window function.

Furthermore, the 3D-PSF and the 3D-TF are a pair of Fourier transformation relations. Therefore, according to the previously reported 3D-PSF model [16,30] based on the angular spectrum method (ASM), it is possible to simulate a PSF without aberrations that conforms to the interference system (the light source wavelength of the CSI system spans from 1.22 μm to 1.42 μm, with an objective NA of 0.5) in this article. The PSF is Fourier transformed to obtain the TF, and the non-zero part of the 3D-TF is extracted as the filtering window function in the frequency domain. The corresponding cross-sectional images of the system PSF, TF, and window function are shown in Figure 6.

**Figure 7:**
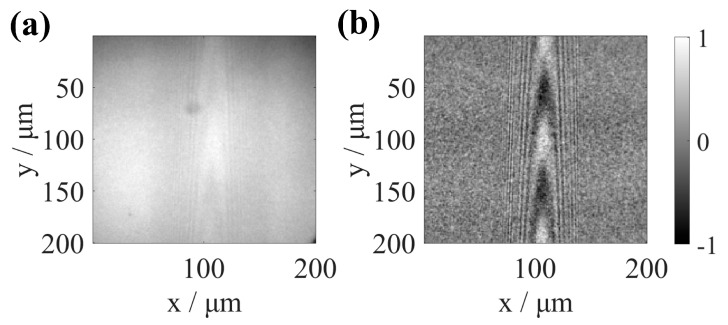
The images (x–y) at the bottom of the trench. (**a**) Original collected interferogram. (**b**) Interferogram after window function processing.

**Figure 8:**
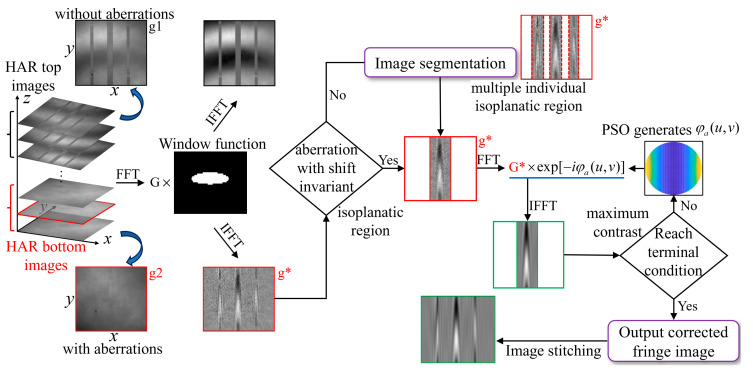
Flowchart of CAO aberration correction based on the PSO algorithm.

During the CSI scanning measurement process, environmental disturbances will superimpose noise on the coherent signal. The non-zero part of the 3D-TF of the microscopic interference system represents the system’s frequency domain bandwidth, and only the information within the bandwidth can be accurately reflected by the system. According to this principle, the 3D interference images captured by the camera are Fourier transformed into the frequency domain. By preprocessing the 3D interference signal with the window function, the frequency information within the bandwidth range of the 3D-TF can be retained. Meanwhile, the low-frequency and high-frequency information outside the TF bandwidth is filtered out, achieving noise reduction in the interference signal. Figure 7b shows the result of the original collected interferogram (Figure 7a) being preprocessed by the window function in the spatial frequency domain. This method effectively suppresses noise and improves the signal-to-noise ratio of the coherent signal. However, the interference fringes of the above signal intensities are insufficient to reconstruct the structure’s actual topography. The interferogram must undergo aberration correction to complete the reconstruction of the structure’s topography. Figure 8 depicts this paper’s numerical processing of interferograms with aberrations, including interferogram preprocessing, image segmentation, and aberration correction. The red dotted line in the red box is the dividing line of the isoplanatic region.

## 3. Experimental Results and Discussions

### 3.1. Aberration Correction for HAR Single Trench

**Figure 9:**
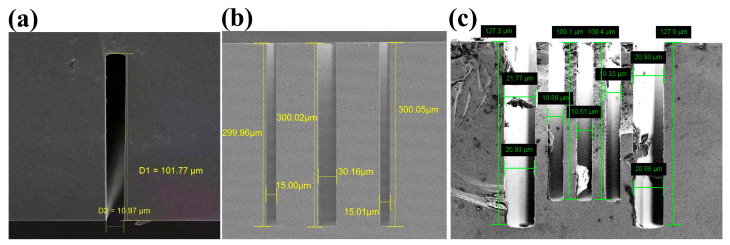
SEM images (x-z section) of trench samples. (**a**) Sample 1. (**b**) Sample 2. (**c**) Sample 3.

**Figure 10:**
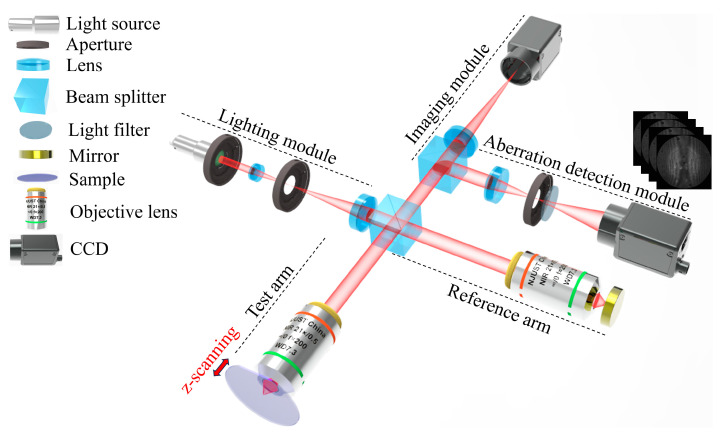
Schematic of near-infrared AC-NIR-CSI.

**Figure 11:**
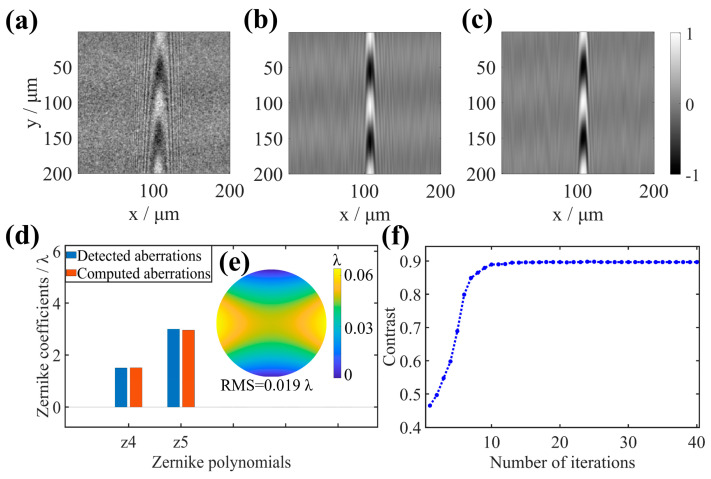
Corrects aberration objects and results. (**a**) Interferogram with aberration. (**b**) Corrected interferogram after PSO–based CAO. (**c**) Interferogram after compensation of aberrations by a deformable mirror. (**d**) Zernike coefficient. (**e**) Phase deviation. (**f**) Interference fringe contrast during the correction process.

This article corrects the aberrations of the interferogram of sample 1 of the single trench HAR as shown in Figure 9 (structural parameters see Table 1). The interferogram is collected using the near-infrared micro-interference (near-infrared AC-NIR-CSI) system shown in Figure 10, with a central wavelength of 1.32 μm and NA of 0.5. The specific design of the system has been reported in [12]. The sample is moved in the optical axis direction (z-axis) with a step size λ/8 to obtain a series of scanned interferograms. Due to the aberrations caused by the HAR structure, the interferogram at the bottom of the HAR structure is severely affected by aberrations, resulting in the poor contrast of interference fringes and fringe distortion. Figure 11a shows the interferogram at the bottom of the trench at the focal position.

Table 1 shows the values of the structural parameters (depth and width) of all the HAR samples involved in this paper, which are measured by SEM system (Model: Crossbeam 350, Manufacturer: Carl Zeiss Microscopy Limited is located in the city of Jena, Germany, the SEM’s measurement uncertainty is within 0.3%).

Figure 11a shows the result of the original interferogram is preprocessed by window function in the spatial frequency domain, and Figure 11b shows the processing result of the interferogram in Figure 11a based on the aberration correction method proposed in this article. The method effectively eliminates the aberrations caused by the sample, and the contrast of the fringes is significantly improved. Figure 11c shows the interferogram at the bottom of the trench by the hardware-based active compensation of aberrations introduced in article [12], and it is processed by a window function in the spatial frequency domain for comparison with the results of the aberration numerical correction method proposed in this paper. The contrast values of the two interferograms (Figure 11b and Figure 11c) are 0.89 and 0.90, respectively, indicating that the aberration processing scheme in this paper can be comparable to the hardware compensation aberration method. Moreover, the aberrations computed using the PSO algorithm are compared with the aberrations detected using the aberration detection module in the near-infrared AC-NIR-CSI system, where the measurement of pupil aberrations is accomplished using a seven-step phase-shifting method. Since the aberrations caused by HAR samples are mainly composed of defocus and astigmatism, only these two aberrations are shown. The defocus and astigmatism coefficients in the Zernike polynomial corresponding to the aberrations are shown in Figure 11d, with the values of each coefficient being basically consistent. According to the PSO algorithm, the calculated defocusing coefficient is 1.6 λ, and the astigmatism coefficient is 3.2 λ. The root mean square (RMS) value of the residual wavefront between the calculated aberrations and the experimentally measured aberrations is 0.019 λ (as shown in Figure 11e). Therefore, the proposed PSO-based CAO algorithm can effectively correct aberrations and accurately calculate the aberrations caused by the sample.

The interference fringe contrast during the PSO algorithm iteration process is shown in Figure 11f. When the iteration reaches fifteen times, the contrast of the interference fringes is stable at the ideal value (0.89 λ). It indicates that the PSO algorithm has the operational advantage of rapid convergence. The calculation time of the method proposed in this paper depends on various factors, such as image size, number of iterations, selected optimization Zernike polynomial terms, and computer hardware configuration. The efficiency analysis of applying the method in this paper to correct the aberrations of the interferogram, as shown in Figure 11a on a Windows machine with Intel^®^ Core™ i7-10700 2.90 GHz processor and 16.0 GB RAM, is shown in Table 2.

**Figure 12:**
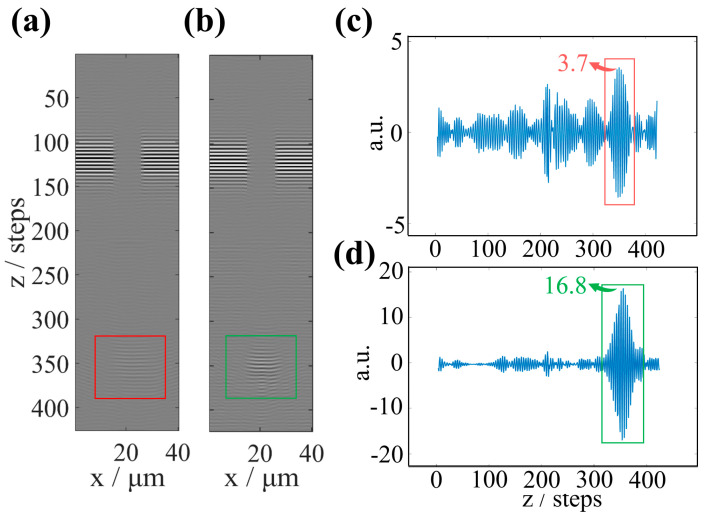
Corrects aberration objects and results. (**a**,**c**) The interference signal with aberrations. (**b**,**d**) The corrected interference signal. (**a**) The x–z cross-section (red box) of the interferogram with aberrations stacked along the optical axis. (**b**) The x–z cross-section (green box) of the interferogram after correction for aberrations. (**c**,**d**) The interference signal at the central sampling point in the structure bottom area.

Figure 12a displays the x-z section of the interferogram with aberrations stacked along the optical axis. The interference fringes at the top of the groove are clear and have high contrast. Due to the influence of aberrations, the contrast of the interference fringes at the bottom of the trench is poor (red box). According to the method presented in this paper, the aberration correction of the interferogram collected along the optical axis is carried out frame by frame. It needs to be stressed that previous research [12] observed that, apart from significant defocus changes resulting from scanning along the z-direction, the variation of sample-induced other aberrations is smaller than half a wavelength; that is, the sample-induced aberrations remain essentially unchanged during trench scanning. Therefore, the Zernike aberration terms and coefficients used to correct interferograms for each frame from scanning along the z-direction are calculated from the HAR structure’s interferogram of the bottom focus position by the algorithm proposed in this paper. It can improve the efficiency of aberration correction.

After aberration correction, the contrast of the interference fringes at the bottom of the trench is significantly improved (green box), as shown in Figure 12b. Figure 12c,d show the interference signal at the center sampling point in the bottom region, and after aberration numerical correction, the interference signal intensity is significantly improved. The signal intensity after aberration correction is 4.5 times that before correction, and the signal restored a single peak, facilitating the 3D topography construction. Therefore, the correction effect of the experimental data further proves the feasibility of the aberration correction method presented in this paper.

**Figure 13:**
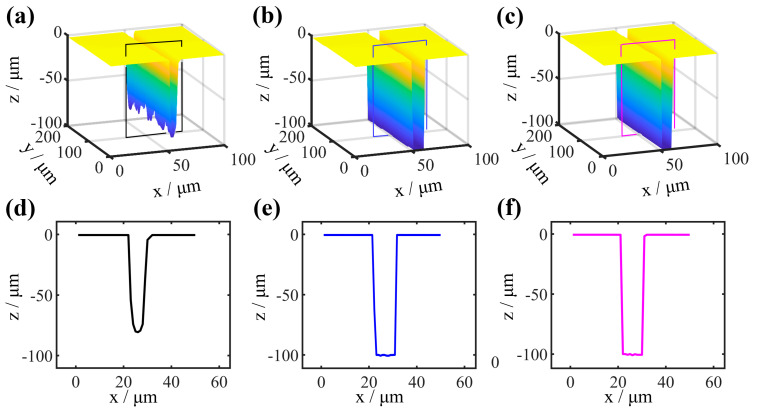
3D topography construction of sample 1. (**a**) 3D topography of uncorrected aberrations. (**b**) 3D topography of corrected aberrations. (**c**) 3D topography after compensation of aberrations by a deformable mirror. (**d**) The x–z cross-section of the topography of (**a**). (**e**) The x–z cross-section of the topography of (**b**). (**f**) The x–z cross-section of the topography of (**c**).

Based on the interference fringes before and after correction, the 3D topography of sample 1 is constructed, as shown in Figure 13. Among them, Figure 13a is the 3D topography constructed based on the original interference signal, and there is a significant difference in depth at the bottom viewpoints, manifested as serrations. The 3D topography after aberration correction is shown in Figure 13b, and the depth at the bottom viewpoints of the structure is consistent. The 3D topography by the hardware-based active compensation of aberrations is shown in Figure 13c. The x-z cross-section of the above three 3D topographies is, respectively, shown in Figure 13d–f. Through the x-z cross-section of the 3D topography, the average value of the structure parameters can be directly calculated after aberration correction. Both the aberration correction method in this paper and the hardware active compensation aberration method can calculated complete the measurement of the structure of HAR samples, the average values of the structural parameters (width and depth) obtained through calculation are close to the SEM measurement values, and the relative error is much less than 1%.

**Figure 14:**
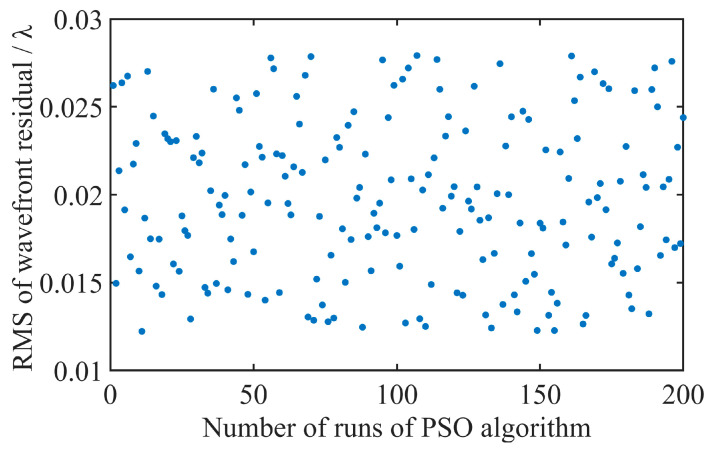
Results of 200 random operations of PSO algorithm.

In addition, to test the robustness of the method proposed in this paper, on the same computer, the interferogram collected by sample 1 is randomly processed 200 times, and the aberrations computed using the PSO algorithm are compared with the aberrations detected using the aberration detection module in the near-infrared AC-NIR-CSI system. The RMS values of the residual wavefront between the calculated aberrations and the experimentally measured aberrations, as depicted in Figure 14, fall within the range of 0.01 λ to 0.03 λ. It is indicated that the computational aberration correction of the method proposed in this paper can accurately predict the wavefront aberration and achieve high-precision aberration correction.

### 3.2. Aberration Correction for HAR Hybrid Trench Array

**Figure 15:**
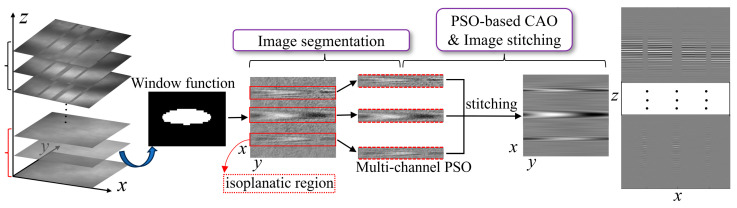
Flowchart of aberration correction process for HAR hybrid trench array.

Both the under-correction and over-correction of wavefront aberration can affect the accuracy of sample topography construction. Therefore, for the shift-variant aberrations caused by the HAR hybrid trench array composed of multiple trenches with different parameters, each trench under test can be considered as a separated isoplanatic region, a multi-channel aberration correction scheme utilizing the virtual phase filter based on isoplanatic region segmentation is established. The multi-channel parallel signal processing method is implemented to achieve accurate and rapid CAO aberration correction for each area. After aberration correction, the weighted fusion method is used to stitch images of each isoplanatic region [33,34,35]; limited by the length of the article, it will not be repeated. The above process is illustrated in Figure 15.

**Figure 16:**
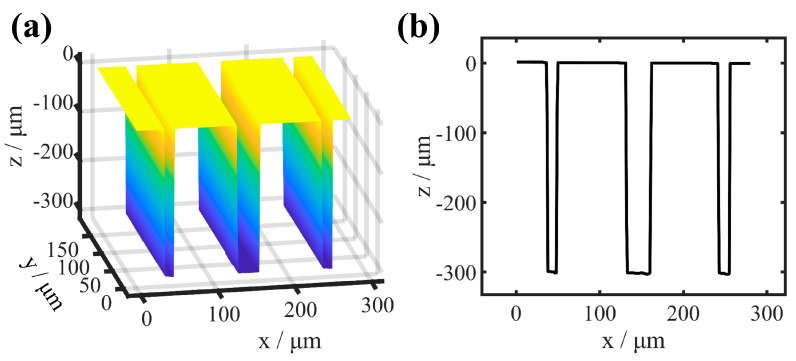
3D topography construction of sample 2. (**a**) 3D topography. (**b**) The x–z cross-section of the topography.

**Figure 17:**
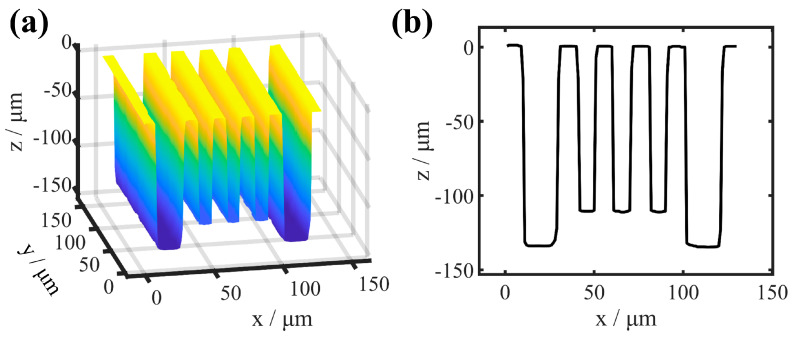
3D topography construction of sample 3. (**a**) 3D topography. (**b**) The x–z cross-section of the topography.

The topography of the hybrid trench array of sample 2 and sample 3 are constructed based on the interferometric signal after aberration correction, as shown in Figure 16 and Figure 17, including three and five single trenches with different parameters, respectively, and these single trenches with depths ranging from 110 μm to 300 μm and widths from 10 μm to 30 μm. The depths and widths of each trench are close to the SEM measurement values in Table 1, indicating that the aberration correction method proposed in this paper is also effective in correcting the shift-variant aberrations introduced by the HAR hybrid trench array samples.

## 4. Conclusions

Due to the significant differences in aberrations introduced by the trenches with different parameters during CSI measurement, the aberrations are shift-variant for the HAR hybrid trench array. To eliminate the impact of aberration on the measurement of surface topography using CSI, we propose a computational aberration correction method for the HAR structure topography measurement based on the PSO algorithm. We set the non-zero part of the 3D-TF as a window function to preprocess the interferogram by filtering out the signals outside the window. Each trench under test can be considered as a separate isoplanatic region, and a multi-channel aberration correction scheme utilizing the virtual phase filter based on isoplanatic region segmentation is established for hybrid trench array samples. For each isoplanatic region, a virtual phase filter constructed through a combination of Zernike polynomials is employed to eliminate the aberration, and the PSO algorithm derives the optimal Zernike polynomial coefficients using interference fringe contrast as the optimization function. The effectiveness and accuracy of the PSO algorithm for CAO can be verified by correcting the aberration in the interferogram of a single trench. For the shift-variant aberrations introduced by two hybrid trench array samples, the interferogram stitching is completed after aberration correction, and the 3D topography of the hybrid trench array is accurately constructed based on the corrected interference signals. This study presents an innovative method for measuring HAR microstructures, which exhibits considerable promise for applications in HAR manufacturing.

## Figures and Tables

**Table 1:** SEM measurement value of the samples.

Single Trench (Sample 1)	Trench		
Depth/μm	101.77		
Width/μm	10.97		
HAR	10:1		
Hybrid trench array (sample 2)	Trench 1	Trench 2	Trench 3
Depth/μm	15.00	30.16	15.01
Width/μm	299.96	300.02	300.05
HAR	20:1	10:1	20:1
Hybrid trench array (sample 3)	Trench 1	Trenches 2/3/4 (mean value)	Trench 5
Depth/μm	21.35	10.61	20.80
Width/μm	127.30	109.25	127.00
HAR	6:1	10:1	6:1

**Table 2:** Efficiency analysis of correcting wavefront aberrations.

Type of Interferogram	Pixel Size	Number of Iteration	Processing Time/s
Interferogram (Figure 9a)	200 × 200	40	5

## Data Availability

The data presented in this study are available upon request from the corresponding author.

## Abbreviations

The following abbreviations are used in this manuscript:

HAR	High aspect ratio
CSI	Coherence scanning interferometry
PSO	Particle swarm optimization
3D-TF	3D transfer function
CAO	Computational adaptive optics
PSF	Point-spread function
ASM	Angular spectrum method
NA	Numerical aperture
FDTD	Finite-difference time-domain
CTF	Coherent transfer function
DC	Direct current
AC	Alternating current
RMS	Root mean square
